# The NADPH Oxidases Nox1 and Nox2 Differentially Regulate Volatile Organic Compounds, Fungistatic Activity, Plant Growth Promotion and Nutrient Assimilation in *Trichoderma atroviride*

**DOI:** 10.3389/fmicb.2018.03271

**Published:** 2019-01-23

**Authors:** Valter Cruz-Magalhães, Maria Fernanda Nieto-Jacobo, Eline van Zijll de Jong, Michael Rostás, Fabiola Padilla-Arizmendi, Diwakar Kandula, Janaki Kandula, John Hampton, Alfredo Herrera-Estrella, Johanna M. Steyaert, Alison Stewart, Leandro Lopes Loguercio, Artemio Mendoza-Mendoza

**Affiliations:** ^1^Bio-Protection Research Centre, Lincoln University, Lincoln, New Zealand; ^2^Department of Biological Sciences (DCB), State University of Santa Cruz (UESC), Ilhéus, Brazil; ^3^UGA-LANGEBIO, Cinvestav, Irapuato, Mexico; ^4^Lincoln Agritech Ltd., Lincoln, New Zealand; ^5^The Foundation for Arable Research (FAR), Christchurch, New Zealand

**Keywords:** *Trichoderma*, plant growth promotion, volatile organic compounds, Nox1, Nox2, NoxR, reactive oxygen species

## Abstract

In eukaryotic systems, membrane-bound NADPH oxidases (Nox) generate reactive oxygen species (ROS) as a part of normal physiological functions. In the soil-borne mycoparasitic and plant facultative symbiont *Trichoderma atroviride*, Nox1 and the regulator NoxR are involved in differentiation induced by mechanical damage, while the role of Nox2 has not been determined. The knock-out strains Δ*nox1*, Δ*noxR* and Δ*nox2* were compared to the parental strain (WT) in their ability to grow and conidiate under a series of stress conditions (osmotic, oxidative, membrane, and cell-wall stresses). All three genes were differentially involved in the stress-response phenotypes. In addition, several interactive experiments with biotic factors (plant seedlings and other fungi) were performed comparing the mutant phenotypes with the WT, which was used as the reference strain. Δ*nox1* and Δ*noxR* significantly reduced the antagonistic activity of *T. atroviride* against *Rhizoctonia solani* and *Sclerotinia sclerotiorum* in direct confrontation assays, but Δ*nox2* showed similar activity to the WT. The Δ*nox1*, Δ*noxR*, and Δ*nox2* mutants showed quantitative differences in the emission of several volatile organic compounds (VOCs). The effects of a blend of these volatiles on plant-growth promotion of *Arabidopsis thaliana* seedlings were determined in closed-chamber experiments. The increase in root and shoot biomass induced by *T. atroviride* VOCs was significantly lowered by Δ*noxR* and Δ*nox1*, but not by Δ*nox2.* In terms of fungistatic activity at a distance, Δ*nox2* had a significant reduction in this trait against *R. solani* and *S. sclerotiorum*, while fungistasis was highly increased by Δ*noxR* and Δ*nox1*. Identification and quantification of individual VOCs in the blends emitted by the strains was performed by GC-MS and the patterns of variation observed for individual volatiles, such as 6-Pentyl-2H-pyran-2-one (6PP-1) and (E)-6-Pent-1-enylpyran-2-one (6PP-2) were consistent with their negative effects in plant-growth promotion and positive effects in fungistasis at a distance. Nox1 and NoxR appear to have a ubiquitous regulatory role of in a variety of developmental and interactive processes in *T. atroviride* either as positive or negative modulators. Nox2 may also have a role in regulating production of VOCs with fungistatic activity.

## Introduction

Reactive Oxygen Species (ROS) are small molecules derived from oxygen, capable of interacting with proteins, lipids, carbohydrates, nucleic acids, and several other small organic molecules (Bedard and Krause, 2007; Hernandez-Oñate and Herrera-Estrella, 2015). ROS function has been associated with the destruction of cellular components as a defense mechanism, although there is increasing evidence that ROS have important roles in various reversible regulatory processes in virtually all eukaryotic cells and tissues (Aguirre et al., 2005; Takemoto et al., 2007; Scott and Eaton, 2008; Livanos et al., 2012; Scott, 2015). Most ROS are produced by NADPH oxidases (Nox family), which transfer electrons from NADPH to oxygen (Bedard and Krause, 2007; Marschall and Tudzynski, 2016), and play a critical role in host defense, posttranslational modification of proteins, cell communication, regulation of gene expression, and cell differentiation (Aguirre et al., 2005; Cano-Dominguez et al., 2008; Hernandez-Oñate et al., 2012; Hernandez-Oñate and Herrera-Estrella, 2015). These features support the general notion that NADPH oxidases are required for organisms’ development (Bedard and Krause, 2007; Kim et al., 2011).

Fungal NADPH oxidases participate in a variety of processes, including plant pathogenicity [e.g., *Magnaporthe oryzae* (Egan et al., 2007), *Botrytis cinerea* (Segmuller et al., 2008), and *Verticillium dahliae* (Zhou et al., 2017)]; sclerotia formation in *Sclerotinia sclerotiorum* (Kim et al., 2011); mutualistic regulation in the *Epichloë festucae* endophyte (Tanaka et al., 2006); mycoparasitism and conidiation in *Coniothyrium minitans* (Wei et al., 2016); secretion of hydrolytic enzymes and biocontrol activity in *Trichoderma harzianum* (Montero-Barrientos et al., 2011); and development of fungal multicellular structures (Kim et al., 2011). NADPH oxidases in fungi vary in number from none to three genes: NoxA/1 and NoxB/2 typically perform the catalytic function in NADPH oxidase complexes (Scott, 2015). Thus far, little is known about NoxC/3 and its function is not clear in fungi. The NoxD/4 subunit interacts with NoxA/1 in the *Botrytis cinerea* model (Siegmund et al., 2015). NoxR has been shown to regulate activation of Nox1/A and Nox2/B (Takemoto et al., 2007; Scott and Eaton, 2008; Tanaka et al., 2008; Marschall and Tudzynski, 2016) as its gene deletion has resulted in phenotypes similar to those of *Δnox1* and *Δnox2* (Cano-Dominguez et al., 2008; Segmuller et al., 2008; Scott, 2015).

Studies regarding specific roles of *Nox* genes point to a species-specific variation of their functions in fungal systems. Genes orthologous to *NoxA/1* participate in germination and pathogenicity in *Claviceps purpurea* (Giesbert et al., 2008) and *Alternaria alternata* (Yang and Chung, 2012). In *Aspergillus nidulans*, deletion of *NoxA* blocks differentiation of sexual fruit bodies (cleistothecia), but does not affect hyphal growth (Lara-Ortiz et al., 2003). Moreover, *Fusarium graminearum NoxA*, but not *NoxB*, is involved in perithecia development and ascospore production (Wang et al., 2014). It has been postulated that the distribution of *NoxA* in the fungal kingdom is strictly correlated with the ability of filamentous fungi to differentiate sexual structures through ROS regulation (Lalucque and Silar, 2003). Nox proteins can also have a role in defense and signaling in cellular differentiation (Takemoto et al., 2007), which is regarded as a conserved function in eukaryotes (Cano-Dominguez et al., 2008). *NoxB/2* is found in most fungi containing *NoxA/1*, and in some instances partial functional overlap has been observed (Tanaka et al., 2006). However, distinct and conserved roles for each of these proteins have also been observed. For example, in *M. grisea, B. cinerea* and *C. purpurea*, NoxA/1 regulates fungal proliferation within plant tissues (Egan et al., 2007; Giesbert et al., 2008; Segmuller et al., 2008), while NoxB/2 is required for plant cuticle penetration in *M. grisea, V. dahliae*, and *B. cinerea* (Segmuller et al., 2008; Ryder et al., 2013; Zhou et al., 2017).

In *B. cinerea*, the BcnoxA and BcnoxD subunits interact with each other and are involved in pathogenicity, fusion of conidial anastomosis tubes (CAT) and formation of sclerotia and conidia (Segmuller et al., 2008; Roca et al., 2012; Siegmund et al., 2015). In other fungal systems, however, Nox2/B appears to be involved in different cellular functions. In *Neurospora crassa*, NOX-2 regulates germination of sexual spores while NOX-1 controls asexual development and hyphal growth (Cano-Dominguez et al., 2008). In *C. purpurea* and *F. graminearum*, the Δ*noxA* and Δ*noxA/*Δ*noxB* mutants do not form perithecia nor ascospores, whereas this phenotype is not observed in Δ*noxB* and only NoxA inactivation affects virulence (Giesbert et al., 2008; Wang et al., 2014). In the endophyte *E. festucae*, the deletion of *NoxA* or *NoxR* genes resulted in a change from mutualistic to pathogenic life style in the fungus but not when *NoxB* gene was deleted (Takemoto et al., 2006; Tanaka et al., 2006). In *T. harzianum*, the overexpression of the *Nox1* gene compromised its mycoparasitic activity against *Pythium ultimum* and upregulated diverse lytic enzymes (Montero-Barrientos et al., 2011). In *Trichoderma atroviride*, NoxR and Nox1 participate in differentiation induced by mechanical injury (Hernandez-Oñate et al., 2012), superoxide accumulation, response to extracellular ATP and phosphorylation of the MAPK Tmk3, which is related to various abiotic stresses (Hernandez-Oñate et al., 2012; Medina-Castellanos et al., 2014), while Nox2 is dispensable for all these processes and its role remains unknown.

*Trichoderma atroviride* is an interesting experimental model, as it has been extensively used as the formulation basis for bio-products applied to agriculture (Woo et al., 2014), providing beneficial effects such as plant growth promotion (Gravel et al., 2007; Salas-Marina et al., 2011) and tolerance to a variety of abiotic and biotic stresses (Woo et al., 2006). In this study, we report how *Nox* mutants (Δ*noxR*,Δ*nox1*, and Δ*nox2*) from *T. atroviride* are affected in the regulation of metabolic pathways and fungal development under different stress conditions. Furthermore, by assessing how these genes participate in biotic interactions with other fungi and plants, we report for the first time a role of Nox2 in synthesis/emission of volatile organic compounds (VOCs). Moreover, we describe the role of VOCs production with fungistatic activity and plant growth promotion emitted by these *T. atroviride* mutants.

## Materials and Methods

### Fungal Material and Growth Conditions

*Trichoderma atroviride* IMI 206040 was used as the wild-type strain (WT). Hernandez-Oñate et al. (2012) previously reported the Δ*noxR*, Δ*nox1*, and Δ*nox2* deletion mutants and the corresponding complementations for Δ*noxR*, Δ*nox1*. *Trichoderma* spores from the different strains were propagated on potato dextrose agar (PDA) (Difco) at 25°C for 7 days in a 12/12 light/dark photoperiod. For *R. solani* RS73-13b (LU8003) propagation, a mycelial disk (5 mm) was inoculated in the center of a PDA plate. For *S. sclerotiorum* (LU8006), mature sclerotia from PDA plates were transferred to the center of fresh PDA plates and grown for 5 days according to Steyaert et al. (2016).

### Complementation Experiments

For genetic complementation of Nox2 deletion mutants, total genomic DNA was extracted from a shaking culture of wild type *T. atroviride* IMI206040 grown at 25°C in PDB medium for 48 h using a phenol: chloroform method and ethanol precipitation. Briefly, the mycelium was recovered using three layers of sterile Miracloth, immediately frozen in liquid nitrogen and grinded using a mortar and pestle. For genomic DNA extraction, 300 mg of mycelium powder were used per every 400 μL of TE-Phenol-Chloroform and 400 μL lysis buffer (2% Triton X100, 1% SDS, 100 mM NaCl, 10 mM Tris-HCl pH 8.0 and 1 mM EDTA). The mixture was vortexed for few seconds and centrifuged at 12,000 × *g* for 8 min at room temperature. Supernatant was recovered and genomic DNA precipitated with pure ethanol; the pellet was washed with 500 μl ethanol 70% and air-dried before dissolved with TE buffer with RNAse.

Genomic DNA from the *T. atroviride* IMI206040 WT was used as template to amplify the *Nox2* gene. By using the publicly available genomic DNA sequence from *T. atroviride*^1^, we manually designed a couple of primers to amplify Nox2 (Protein ID:300495 in JGI database) plus the regions corresponding to the promoter and terminator, as indicated below. Phusion High-Fidelity Taq Polymerase (Thermo Fisher Scientific Inc., MA, United States) and the primer combination oAM-LU81 (5′-GGAGAGCAAAGGCGGGAAATG-3′) and oAM-LU84 (5′-AGCTGCAATACGAGCAGGTTTC-3′) were used to amplify a 5.48-Kb fragment containing the Nox2 open reading frame, 2.3-Kb upstream sequence region and 1.28-Kb of downstream sequence.

The PCR amplification was carried out in 50 μl reactions containing 1 μl (100 ng) of genomic DNA template, 10 μl of 5X Phusion HF Buffer, 1 μl dNTPs (10 mM stock), 1.5 μl DMSO, 1 μl each primer (10 μM stock), 0.5 μl Phusion^®^High-Fidelity DNA Polymerase (2 units/mL) and 34 μl of de-ionized water (ddH_2_0). All reactions were run on a Bio-Rad iCycler thermal cycler. *Nox2* was amplified using the following parameters: initial denaturation at 98°C for 1 min, 35 cycles of 98°C for 35 s; 60°C for 10 s and 72°C for 3 min, followed by a final extension at 72°C for 10 min. PCR product was subsequently A-tailed using MangoTaq DNA Polymerase (Bioline Reagents Ltd., London, United Kingdom) before being cloned into the pCR-XL-TOPO vector (Invitrogen, Thermo Fisher Scientific, MA, United States) following the manufacturer’s specifications. The resulting plasmid was called pCR-XL-TOPO-Nox2. The mutated version of *tub2* gene from *Trichoderma viride*, which confers resistance to the fungicide benomyl (Goldman et al., 1993), was integrated into the single *Not*I restriction site in the pCR-XL-TOPO-Nox2 plasmid. The resulting plasmid (pCR-XL-TOPO-Nox2-tub2) was linearized with *Sma*I and used to transform protoplasts of the Δ*nox2* strain using the protocol described by Baek and Kenerley (1998). The colonies were selected in 2.5 μg of benomyl (Sigma-Aldrich, Merck KGaA, Darmstadt, DE) per mL of PDA-S (0.7% agar and 0.5 M sucrose). Five rounds of spore isolation were conducted on PDA supplemented with 2.5 μg of benomyl.

To confirm the nox2 complementation in Δ*nox2* mutants, a PCR reaction using around 100 ng genomic DNA from the wild type, Δ*nox2* mutants and nox2 complementing strains (transformed with the pCR-XL-TOPO-Nox2-tub2 vector) were used as templates. Nucleotides corresponding to the position 767 to 1306 from the ATG of *nox2* gene were amplified by PCR using primers oAM-LU324 (5′- ACCACATGTTCATCATCTTC-3′) and oAM-LU366 (5′-CTGATCCGAAGGGGCCGTCG-3′). The PCR was performed using Faststart Taq polymerase (Roche Biochem Reagents, Mannheim, Germany) following the specifications of the manufacturer. The PCR conditions were as follow: initial denaturation/activation of polymerase at 95°C for 5 min, 35 cycles of 95°C for 30 s; 60°C for 30 s and 72°C for 1 min, followed by a final extension at 72°C for 7 min. The PCR products were analyzed in 0.8% agarose TAE gel. The return of complemented strains to WT phenotypes was observed (Supplementary Figure S1).

### Growth and Conidiation Under Stress Conditions

Fungal inocula consisted of conidia harvested from 7 day-old cultures of *T. atroviride* grown on PDA plates at 25°C under a 12/12 light/dark photoperiod. Conidia were collected in sterile bi-distilled water (SBW), filtered through two layers of Miracloth (Calbiochem^TM^, Merck KGaA, Darmstadt, DE) to eliminate mycelia, counted in a hemocytometer and adjusted to 1 × 10^6^ spores in 5 μL SBW.

To analyse the sensitivity to different stress conditions, 1 × 10^6^ spores of the WT, Δ*noxR*, Δ*nox1* and Δ*nox2* mutants were inoculated on the center of PDA plates. For osmotic stress, PDA was supplemented with NaCl (0.6 M), or sorbitol (1.0 M); for membrane stress, the medium was supplemented with SDS (0.014%); for cell-wall stress, calcofluor white (600 μg/mL) and Congo red (1000 mg/mL) were used separately. For oxidative stress, menadione (0.5 mM) was chosen. Plates without the stressors were used as control. Plates were incubated at 25°C for 7 days in a 12/12 light/dark photoperiod. The colony diameter was measured every 24 h and at the end of the experiment the total number of conidia produced was determined by counting in hemocytometer. Six replicates were set up for each strain and each stress condition. Experiments were repeated two times.

### Dual Culture of Mutants With Plant Pathogenic Fungi

Antagonism of *T. atroviride* WT and mutants against *R. solani* and *S. sclerotiorum* was assessed by an *in vitro* dual-culture method as reported previously by Steyaert et al. (2016). Briefly, a mycelial plug from the colony margin of an actively growing *Trichoderma* culture was placed 2 cm away from the border of Petri dishes with PDA medium. After 24 h, a plug of the plant pathogen (*Rhizoctonia solani, Sclerotinia sclerotiorum*) was similarly placed 2 cm away from the edge of the Petri plate but on the opposite end of the corresponding *Trichoderma* strain (WT, Δ*noxR*, Δ*nox1*, or Δ*nox2*). The plates were sealed with Millipore tape and incubated at 25°C under 12/12 h light/dark photoperiod. Observations of growth and inhibition characteristics were made every 24 h and images of the confrontation assays were taken to record the interaction. The final evaluation was performed when the plant pathogen completely covered the control plate without *Trichoderma*. Percentage inhibition was calculated by the formula: Inhibition (%) = ([C - T) × 100]/C, where C = fungal diameter (cm) in the control plate, and T = fungal diameter (cm) in the *Trichoderma*-treated plates (Steyaert et al., 2016). The experiment was conducted twice with three repetitions for each *Trichoderma* strain (treatment).

### Effect of *Trichoderma* Volatile Organic Compounds (VOCs) on *Arabidopsis thaliana* Growth

The effect of *Trichoderma* VOCs on *A. thaliana* was previously described (Nieto-Jacobo et al., 2017). Briefly, five surface sterilized seeds of *A. thaliana* (Col-0) were placed onto one side of a 90 mm diameter double compartment Petri plate (Thermo Scientific Labserv, NZ) containing 0.2 × MS medium (Murashige and Skoog basal salt mixture, catalog M5524, Sigma-Aldrich, Merck KGaA, Darmstadt, Germany) supplemented with 0.6% sucrose and adjusted to pH 7.0. Plates were sealed with plastic film and placed on their edge at an angle of approximately 65° and incubated at 22°C under 16 h light/8 h dark regime. After 7 days, 5 μL of a *Trichoderma* spore suspension (containing 1 × 10^6^ spores) was inoculated into the opposite compartment on PDA medium and the plates were re-sealed and incubated for an additional 7 days. Fresh weight of shoots, roots and total biomass per plant were measured. Shoots or roots of 10 plants were pooled and biomass was measured, being then divided by the number of plants to express the weight per plant. Four plates per strain were analyzed each time. The experiment was carried out three times. Experiments to assess the role of VOCs and carbon dioxide were conducted using the treatments suggested previously (Piechulla and Schnitzler, 2016). These experiments were conducted as mentioned above, except that immediately after *Trichoderma* inoculation, the plates were placed in big square plates (12 × 12 cm) containing four different treatments: (1) cotton rolls saturated with sterile water (control); (2) cotton rolls saturated with 0.1 M BaOH to reduce the levels of CO_2_; (3) plates as above but with cotton rolls used as control for charcoal plates; and (4) plates with around 4 g of activated charcoal to absorb the VOCs. For the open system (Petri dishes without a lid), plates were placed in sealed plastic containers (1 L) to prevent moisture loss. The seedlings were collected and measured as described by Nieto-Jacobo et al. (2017).

### Effect of *Trichoderma* VOCs on Fungal Plant Pathogens

The effect of VOCs from *T. atroviride* WT and mutants on the growth of *R. solani* and *S. sclerotiorum* was determined using the method described by Steyaert et al. (2016). Briefly, the center of Petri plates (90 mm × 25 mm) containing PDA medium were inoculated with a 5-mm diameter PDA plug excised from the margin of a 3 day-old *T. atroviride* culture and incubated for 48 h at 25°C in a 12 h light/12 h dark photoperiod. Two days after *Trichoderma* inoculation, additional PDA plates of same size were inoculated with a 5-mm PDA plug from the margin of *R. solani* or *S. sclerotiorum* colonies, previously grown for 72 h. The lid from the *Trichoderma* plate was replaced by the PDA plate inoculated with the plant pathogen, in such a way that both fungi were facing each other. The plates with phytopathogenic fungi were placed on top to avoid contamination by conidia of *Trichoderma*. Both plates were sealed with a triple layer of plastic film to prevent the loss of volatile substances. Petri-dishes containing PDA without the antagonist served as control. The plates were incubated at 25°C in a 12 h/12 h light/dark regime for 7 days and the reduced diameter of growth of the plant pathogens (fungistatic activity) was recorded. After 7 days, the volatile treatment ended by replacing the *Trichoderma* plates with sterile lids. The recovery of pathogens’ growth was assessed after an additional 7 days in the same incubation conditions described above. Three independent experiments with five plates per strain were conducted.

### Headspace Analysis of *Trichoderma* VOCs

The VOC profiles of *T. atroviride* WT and mutants were analyzed using a Shimadzu GC-MS QP2010 (Shimadzu Corporation^TM^, Kyoto, JP) gas chromatograph - mass spectrometer fitted with a Restek Rxi-5ms fused silica capillary column (30.0 m × 0.25 mm i.d. × 0.25 μm, Bellefonte, PA, United States) following the specifications described by Nieto-Jacobo et al. (2017).

### Biolog Phenotype Microarray Analysis

The assimilation profile of carbon sources of *T. atroviride* and mutants was investigated using a Biolog FF MicroPlate^TM^ (Biolog, Inc., Hayward, CA, United States). The experiments were conducted as previously reported by Friedl et al. (2008) with the following modifications: conidia were suspended in sterile Biolog FF inoculating fluid (Biolog, Inc., Hayward, CA, United States), mixed in a vortex and adjusted to OD_600 nm_ of 0.2. Aliquots of 100 μL of the conidial suspension was dispensed into each of the wells of the FF MicroPlates (Biolog, Inc., Hayward, CA, United States). The plates were incubated at 25°C under a 12 h/12 h light/dark regime. The optical densities OD_750_ (mycelial growth) and OD_490_ (mitochondrial activity) were measured after 24, 48, 72, 96, and 120 h using a microplate reader (Multiskan GO, Thermo Fisher Scientific^TM^, MA, United States). The OD_750_ at 120 h was chosen as a reference time point for the mycelial growth assays, since it allowed a comparison of biomass formation on all carbon sources. The experiments were completed in independent triplicates. To quantify conidiation, a nominative scale proposed by Friedl et al. (2008) was used. This scale differentiates between the complete absence of conidiophores and conidia (score 0), the formation of immature pustules without mature conidia (score 1), and four levels of conidiation intensity, from weak diffuse sporulation (score 2) up to the development of a conidial coverage in the whole well (score 5). The levels were identified by visual examination of the plate with help of the illuminator of a colony counter (Suntex Instruments Co., EN). This method was used to analyze both carbon and nitrogen sources according to their growth and conidiation profiles.

### Bioactivity Assays of Commercial 6-Pentyl-2H-Pyran-2-One in *R. solani* Growth

Commercial 6-pentyl-2H-pyran-2-one (≥96%, CAS no. 27593-23-3) (6PP-1) was evaluated for bioactivity against *R. solani* in the inverted plate assays. The inverted plate assays were carried out as described above [see section “Effect of *Trichoderma* Volatile Organic Compounds (VOCs) on *Fungal Plant Pathogens*”], except that the 6-pentyl-2H-pyran-2-one was introduced at the time of pathogen inoculation in place of *Trichoderma*. Different concentrations of the 6-pentyl-2H-pyran-2-one (0.32, 1.6, 8, 40, 200, and 1000 μmol) were applied to an antibiotic assay disks (13-mm diameter, Whatman, GE Healthcare Life Sciences, CHI, United States), with the exception of the highest concentration, which was applied in four disks. The disks were adhered around the center of a Petri dish lid with dichloromethane (5 μL/disk, Applied Biosystems, Foster City, CA, United States). The Petri dish with pathogen was placed inverted over the disks and sealed with five layers of plastic film. The positive control was exposed to 50 μL of hexane.

### Statistical Analysis

The data of all but the Phenotype Microarrays experiments were separately subjected to analysis of variance (ANOVA) and means were compared with Fisher’s unprotected least significant difference test (*P* < 0.05) using GenStat 16th edition. Phenotype Microarrays were evaluated by cluster analysis using R software (R Core Team, 2016).

For the plant pathogens growth, the mean fungal diameter was recorded for the pathogens as described above. ANOVA was performed for each experiment using the base 10 logarithm of the mean fungal diameters of the positive control over the treatment.

## Results

### Nox Proteins Regulate *T. atroviride* Morphogenesis Under Diverse Stress Conditions

**FIGURE 1:**
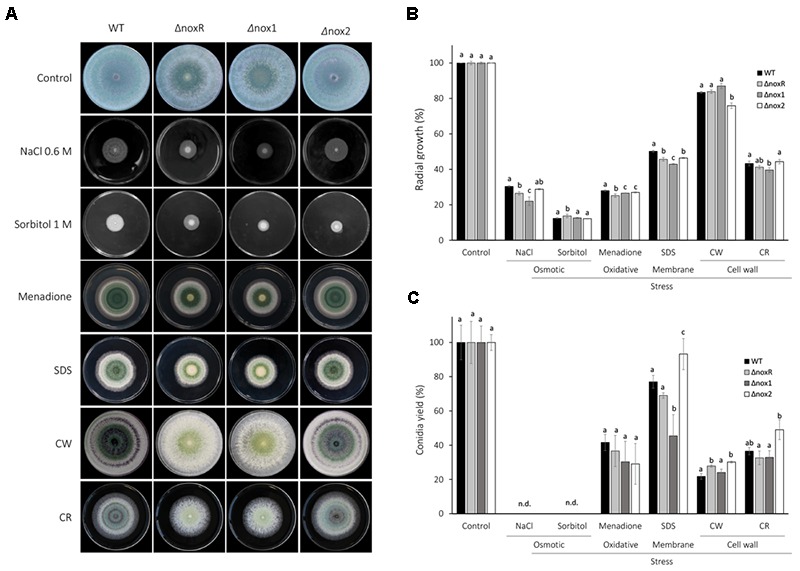
Stress response of *T. atroviride nox* mutants. **(A)** 5 μl containing 1 × 10^6^ conidia were inoculated to the center of PDA plates, supplemented with the indicated stressors (see Methods for concentrations), and incubated at 25°C for 4 days. **(B)** The graphs display the relative values of mycelial growth, and **(C)** conidiation on a percentage scale. The mycelial growth was assessed as an inverse function of the growth speed, which was estimated by the number of days that a colony took to reach the edge of the plate; the highest of these inverted values was assigned as ‘100%’ and the remaining values were set as a fraction (%) of it. For conidiation, the conidia were collected and counted after 7 days of incubation at 25°C; the highest number of spores per treatment was set as ‘100%,’ with the remaining counts set as a fraction of it. The plotted values are the mean of three plates; the experiment was repeated three times with the same results. Statistics was based on Fisher’s test at 5% probability; different letters on top of bars indicate statistically significant differences among strains within each treatment of stressing agent. SDS, Sodium dodecyl sulfate; CW, Calcofluor White; CR, Congo Red.

To verify the major phenotypic effects of single mutations on the three NADPH oxidase genes of *T. atroviride*, the wild-type (WT), Δ*noxR*, Δ*nox1*, and Δ*nox2* strains were assessed for growth and conidiation under osmotic, oxidative, membrane and cell-wall stress conditions. In qualitative terms of colony and conidiation morphologies, the Δ*noxR* and Δ*nox1* strains showed almost identical patterns between them, but distinct from the WT, for all conditions tested (Figure 1A). The Δ*nox2* mutant, on the other hand, exhibited colony morphologies very similar to those of the WT in all treatments, except for the 1 M sorbitol (osmotic-stress agent), in which Δ*nox2* was more similar to the other two mutants. Also, Δ*nox2* was morphologically distinct from the other three strains for the Congo Red (CR) stress (Figure 1A).

In quantitative terms relative to control treatments, mycelial mass of all strains showed a >70% decrease under osmotic and oxidative stresses, and ∼50% decrease for cell-membrane stress (Figure 1B). For the cell-wall stressors, two distinct effects were observed: while CR led to a strong decrease in mycelial mass of ∼60% for all strains, Calcofluor White (CW) displayed only a mild decrease of ∼10–15% for most strains, with only Δ*nox2* showing a significantly lower level than the other strains (20% decrease; Figure 1B). Sorbitol caused the greatest decrease in colony growth (mycelial mass) for all strains, and for the two osmotic-stress agents (NaCl and sorbitol), no conidiation was detected (Figure 1C). Overall, all mutants showed either no difference or significantly lower levels of mycelial growth than WT for all treatments, with the exception of sorbitol, for which the Δ*noxR* showed a significantly higher amount of growth (Figure 1B). Differently to what the morphologies displayed (Figure 1A), Δ*noxR* and Δ*nox1* showed differences in radial growth in four out of six stress conditions analyzed (Figure 1B). For instance, Δ*noxR* was more resistant than Δ*nox1* to osmotic and membrane stresses, less resistant to oxidative stress, and showed no difference for cell-wall stresses. Both Δ*noxR* and Δ*nox1* were distinct from the WT for all conditions tested, with exception of cell-wall stresses (both mutants were similar to WT for CW stress, and only Δ*nox1* was different for CR stress; Figure 1A). Interestingly, the three cell membrane and wall stressors (SDS, CW and CR) resulted in a significantly higher conidiation for Δ*nox2* than for all other strains, including WT, except in relation to Δ*noxR* in the cell-wall stress by CW (Figure 1C).

### Nox Proteins Differentially Regulate Carbon Source Assimilation in *T. atroviride*

*Trichoderma atroviride* growth is influenced by reactive oxygen species (ROS) in a nutrient dependent manner (Friedl et al., 2008). Hence, we assessed the influence of Nox proteins on *T*. *atroviride* growth on 95 carbon sources using the FF MicroPlate^TM^ Carbon Sources Biolog plates. The overall usage profile of carbon sources was assessed in terms of mycelial growth and sporulation for all strains (Supplementary Figure S2). Some characteristic features were observed. First, there was greater variation of phenotypes among the strains for mycelial growth than for conidiation, as shown by the greater number of clusters for the former (Supplementary Figures S2A,B), considering all C sources tested. Second, as seen by the dendrograms, the growth and conidiation profiles of Δ*noxR* and Δ*nox1* were more similar to each other for all nutrient sources. Third, the profiles of Δ*nox2* and WT were also more similar to each other, although this was more noticeable for conidiation than for growth. Fourth, Δ*noxR* showed the least amount of conidiation for ∼93% of all sources tested (Supplementary Figure S2).

**FIGURE 2:**
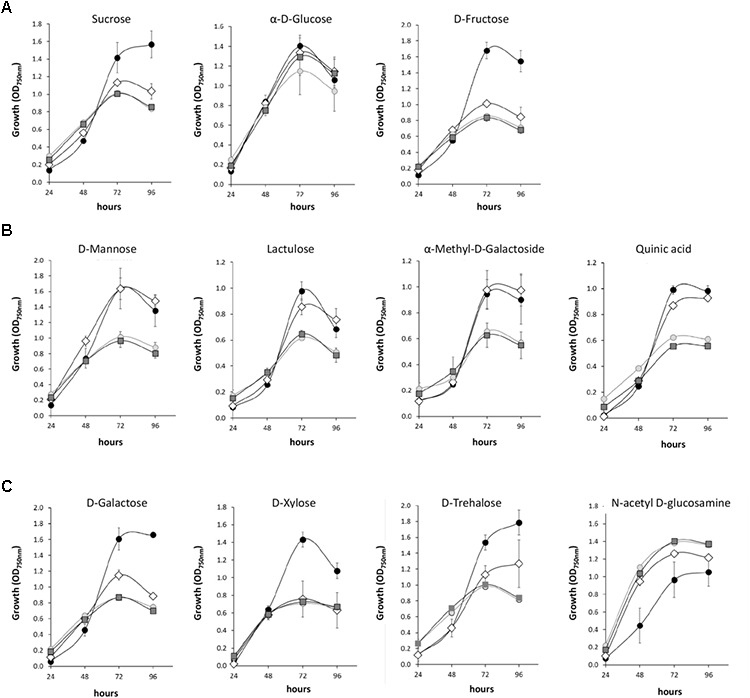
Growth curves of *Trichoderma atroviride* (WT and Nox1, Nox2, and NoxR mutants) on different carbon sources. **(A)** Growth curves on the disaccharide Sucrose and the monosaccharides α-D-Glucose and D-Fructose. **(B)** Growth curves on individual carbon sources where Δ*nox1* and Δ*NoxR* growth are reduced in comparison to the WT and Δ*Nox2*. **(C)** Growth curves on individual carbon sources where the three Nox mutants growth differentially to the WT. Growth was determined based on the *A*_750 nm_ value for the times indicated in the graphic. Standard deviations are given by error bars. The average represented the experiment of three biological replicates. Wild type is indicated by a black circle (●), *ΔnoxR* by a lighter gray circle (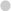), *Δnox1* by a darker gray square (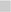) and *Δnox2* strain by an open rhomb (◊).

The WT and mutant strains were also compared for some specific carbon sources assessed individually (Figure 2). The growth of the three mutants (Δ*nox1*, Δ*noxR* and Δ*nox2*) was reduced in sucrose when compared to the WT (Figure 2A). Similar behavior was observed in these mutants when using D-fructose but not α-D-glucose as the carbon source (Figure 2A). The three *Nox* mutants also had reduced growth when D-galactose, D-xylose, or D-trehalose were the carbon sources when compared to the WT strain (Figure 2C). However, the WT and Δ*nox2* grew at similar rates in the D-mannose, lactulose, D-methyl-D-galactoside and quinic acid carbon sources, while Δ*nox1* and Δ*noxR* behaved similarly to each other in the same sources but with lower growth rates than the other two (Figure 2B). Interestingly, when *N*-acetyl D-glucosamine was used as the carbon source, the three mutants performed better than the WT (Figure 2C).

### Nox1 and NoxR Regulate the Direct Interaction Between *T. atroviride* and the Plant Pathogens *R. solani* and *S. sclerotiorum*

**FIGURE 3:**
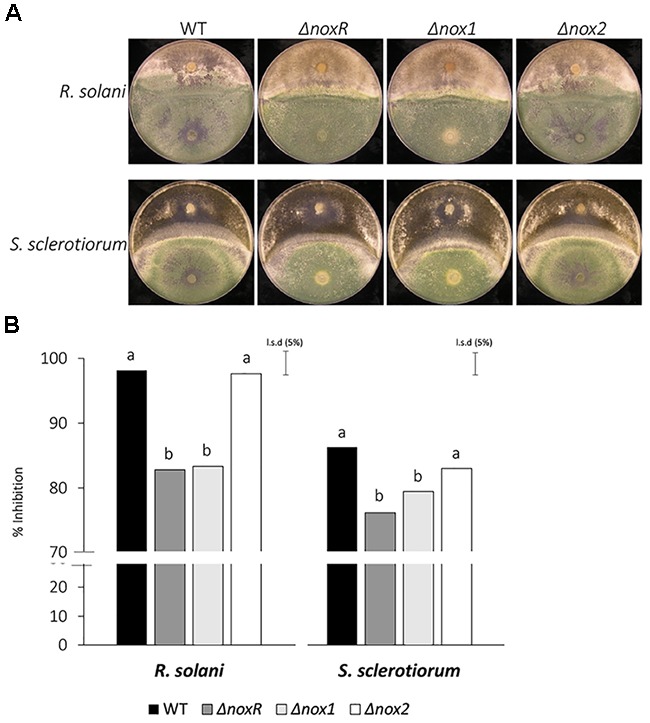
Confrontation assays of *T. atroviride Nox* mutants against *R. solani* and *S. sclerotiorum*. **(A)** The mycoparasitic behavior of *Trichoderma Nox* mutants and WT was assessed on PDA plates, at 25°C for 7 days. *Trichoderma* strains appear at the lower half of the dual-culture plates, whereas the pathogens (indicated on the left) appear on the upper half. **(B)** The inhibition rates were calculated from three replicates, and analyzed by ANOVA using the unprotected Fisher’s LSD test. Different letters on the top of each bar indicate statistically significant differences at *P* < 0.05. The experiment was repeated twice with similar results.

Previous studies have shown that Nox1 from *T. harzianum* is involved in the regulation of antagonism against other fungi (Montero-Barrientos et al., 2011). Here, we were interested in verifying whether the *Nox* mutants from *T. atroviride* could also show an alteration in the antagonistic phenotype by testing dual growth in culture with two phytopathogens, *R. solani* and *S. sclerotiorum* (Figure 3A). The percentage inhibition by WT and Δ*nox2* did not differ and both were significantly greater than those of the Δ*noxR* and Δ*nox1* mutants, for both pathogens (Figure 3B). Interestingly, the shapes of the confrontation edge between *T. atroviride* and the two pathogens were different, reflecting the difference in the antagonistic relationship between them. Whereas for *R. solani* the confrontation line was closer to a straight line, for *S. sclerotiorum* it appeared as a curved line (Figure 3A); by the same token, the straight line for *R. solani* corresponded to a higher inhibition by the *T. atroviride* strains, whereas the curved line (enclosing the *Trichoderma*) indicated a lower inhibition of *S. sclerotiorum* (Figure 3B).

### Nox Proteins Regulate the Production of Plant-Growth Promoting VOCs in *T. atroviride*

**FIGURE 4:**
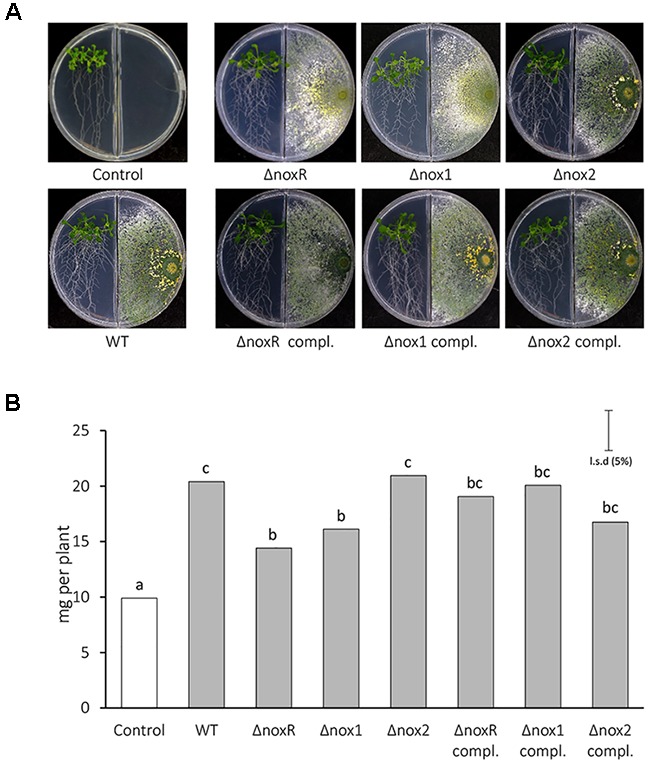
*Arabidopsis* growth promotion induced by VOCs from *T. atroviride Nox*-mutants. **(A)** Morphology of 15-day old seedlings of *A. thaliana* co-inoculated or not with *Trichoderma* in double compartment plates. Medium in both compartments was 0.2X MS medium supplemented with 0.6% sucrose. **(B)** Plant total biomass quantification. A total of five seedlings per plate and three replicates per treatment were used. Total biomass values were analyzed by ANOVA using the unprotected the Fisher’s LSD test. Different letters on the top of each bar indicate statistically significant differences at *P* < 0.05. The experiment was repeated twice with similar results.

Volatile organic compounds are regarded as fundamental infochemicals in microbe-environment interactions (Schmidt et al., 2015) and their emission can be triggered by different abiotic and biotic cues (Polizzi et al., 2011; Bitas et al., 2013; Hung et al., 2013, 2014; Lee et al., 2015, 2016; Nieto-Jacobo et al., 2017). Here, co-culture experiments in closed chambers were performed to test whether the *Nox* mutants could affect the blend of VOCs involved in this plant-microbe interaction, especially in terms of plant-growth promotion (Figure 4). *T. atroviride* was capable of synthesizing/emitting VOCs with plant growth-promotion ability, since all WT and mutant strains produced an increase in the total biomass of *A. thaliana* seedlings, when compared to the control without the fungus (Figure 4A). The total biomass of *A. thaliana* seedlings treated with VOCs from WT did not differ statistically from the effect of VOCs from Δ*nox2*, but Δ*nox1* and Δ*noxR* treatments resulted in a significantly lower rate of biomass increase compared with WT and Δ*nox2* (Figure 4B). These results showed that, in the presence of a functional *Nox2* gene but absence of either *Nox1* or *NoxR, T. atroviride* produces VOCs with inhibitory effects on plant growth. The Nox-mutants supplemented with a fully functional form of the respective mutated genes mostly recovered the WT phenotype in terms of VOCs-inducing plant growth promotion (Figure 4). To assess the influence of carbon dioxide (CO_2_) and VOCs generated by the fungi and the plants in plant growth promotion, we conducted experiments as suggested by Piechulla and Schnitzler (2016) to remove the CO_2_ using 0.1 M Ba(OH)_2_ or removing VOCs by using activated charcoal. As it is observed in Supplementary Figure S3, both CO_2_ and *Trichoderma* VOCs have a significant impact in the promotion of *A. thaliana* growth. Taken together, these results confirmed that part of the observed effects from *Trichoderma* WT and mutants on the growth of *Arabidopsis* seedlings was due to VOCs.

### Nox Proteins Differentially Regulate Fungistatic VOCs Activity in *T. atroviride*

**FIGURE 5:**
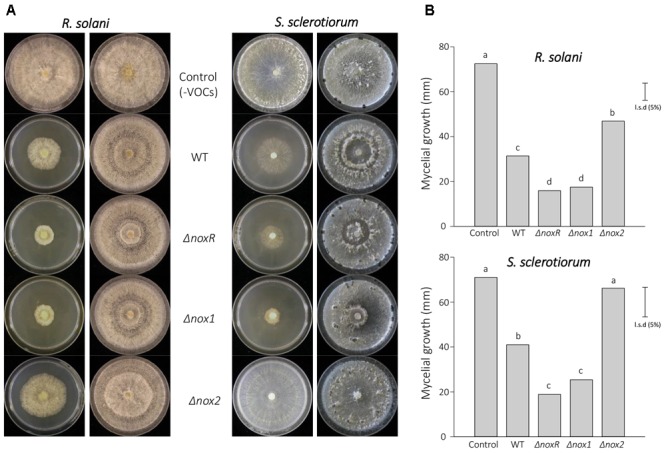
Fungistatic activity against *R. solani* and *S. sclerotiorum* by VOCs from *T. atroviride Nox*-mutants. **(A)** Morphological aspect of *R. solani* and *S. sclerotiorum* in the absence or presence of VOCs emitted by *Trichoderma* strains. Plates in the left columns represent fungal mycelial growth of the pathogens incubated for 4 days at 25°C in the presence of VOCs emitted by *Trichoderma* strains (the absence of the fungus was used as control). Plates in the right columns are the same pathogenic cultures after *Trichoderma* strains were removed, with growth allowed for an additional 10 days. **(B)** The mycelial growth values in the graphs were calculated from four biological replicates, and analyzed by the unprotected Fischer’s test (*P* < 0.05), allowing to infer about the inhibition rates; different letters on top of bars indicate statistically significant differences among strains within each treatment of confronting phytopathogen. The experiment was conducted twice with similar results.

Since the blend of VOCs acting on plant growth promotion could also include other fungistatic compounds acting at a distance (El-Hasan et al., 2007; Lee et al., 2016), we assessed this latter feature in paired-plates with the *T. atroviride* WT and mutant strains facing *R. solani* and *S. sclerotiorum* isolates (Figure 5A). The WT strain demonstrated ability to synthesize/emit VOCs that inhibited growth of both pathogens; when the chamber was opened and the plate with WT strain was removed, the pathogens recovered and reassumed regular growth (Figure 5A), thereby confirming that the observed inhibitory effects were caused by the action of VOCs produced by the *T. atroviride* strain. When the pathogens, however, were subjected to common-chamber cultivation with the *Nox*-mutant strains, the inhibition of the pathogens’ growth differed. For Δ*noxR* and Δ*nox1* strains, the observed VOC-dependent inhibitory effects were stronger than those shown by the WT. Surprisingly, for the Δ*nox2* strain, VOCs production with fungistatic activity was significantly suppressed, thereby allowing higher or normal pathogen growth *in vitro* (Figure 5B). These results showed that, in the presence of a functional *Nox2* gene, but absence of *Nox1* or *NoxR, T. atroviride* produces VOCs with fungistatic activity; however, in the absence of a functional *Nox2*, this function appeared to be severely affected in the interaction with *R. solani*, or almost entirely eliminated in relation to *S. sclerotiorum* (Figure 5A). Similar to the plant growth-promotion experiments, all mutant strains complemented with functional copies of their respective knocked-out genes were able to recover the WT phenotype (data not shown).

### Emission Patterns of Specific VOCs Match to Effects on Plant Growth and Fungistatic Activity in *T. atroviride*

Considering the effects of VOCs observed in the plant growth-promotion (Figure 4) and in the fungistasis at a distance (Figure 5), we assessed the overall profile of volatile compounds produced by WT, Δ*nox1*, Δ*nox2*, and Δ*noxR* strains using headspace solid phase microextraction and GC–MS system (Table 1). All strains produced a blend of 26 to 28 detectable volatiles, with two of them being proportionally more abundant than all others: 2-heptanone (C7-1) and 6-pentyl-2H-pyran-2-one (6PP-1). The differences in total amounts of VOCs among WT and the mutants (Table 1) were not statistically significant. Δ*noxR* and Δ*nox*2 produced on average ∼22% less 2-heptanone than the Δ*nox1* and WT strains. In the case of 11 volatiles, Δ*nox1* and Δ*noxR* produced higher amounts than the WT (Table 1). Based on the pattern of results of plant growth (Figure 4) and fungistatic activity (Figure 5), we focused our analysis on those volatiles displaying similar amounts detected for Δ*nox1* and Δ*noxR* (those with lower plant growth and higher fungistatic effect) but higher in relation to the WT (intermediate fungistatic effect), which was higher in relation to the Δ*nox*2 strain (the lowest to null fungistatic effect). With such pattern of VOCs detection, we identified 8 volatiles in the blend: 2-nonanone, 2-undecanone, (E)-β-farnesene, alpha-bergamotene, 6-pentyl-2H-pyran-2-one, 6-pent-1-enyl-2H-pyran-2-one, and unknown diterpene-1 and -6 (Table 1; italicized). For this set of volatiles matching the fungistasis results (Figure 5), their amounts were not significantly different among strains only for the first three in the list above. Δ*nox2* appeared to have a significantly reduced production for the other five volatiles, especially for 6PP-1 and 6PP-2, whose production was only around 3–4% of that produced by the WT (Table 1). For 3 further volatiles in the blend showing statistically significant difference among the strains (2-heptyl furan, and unknown diterpenes -3 and -4), their levels were either not precisely matching the fungistasis phenotypes (Figure 5) and/or present at insignificant amounts (Table 1).

**Table 1:** Volatile organic compounds (VOCs) production from *wt*, Δ*noxR*, Δ*nox1*, and Δ*nox2* of *Trichoderma atroviride* strain IMI206040.

No.	Class^1^	Compound	Rel. peak area (1 × 10^6^)			
			*wt* (*106.02*)	Δ*NoxR* (*123.79*)	Δ*Nox1* (*183.61*)	Δ*Nox2* (*55.73*)
1	OT-1	2-Methyl-1-butanol^3^	1.94 ± 0.05	2.22 ± 0.35	1.48 ± 0.73	3.45 ± 0.53
2	C7-1	2-Heptanone^2^	42.80 ± 1.72	33.40 ± 5.17	41.60 ± 1.35	33.70 ± 1.43
3	C7-2	2-Heptanol^2^	4.18 ± 1.25	2.61 ± 1.14	2.62 ± 0.19	1.97 ± 0.57
4	C8-1	1-Octen-3-ol^2^	0.30 ± 0.16	0.32 ± 0.22	0.10 ± 0.03	0.49 ± 0.20
5	C8-2	3-Octanone^2^	2.35 ± 0.44	2.79 ± 1.27	1.13 ± 0.11	5.00 ± 1.06
6	FU-1	2-Pentyl furan^2^	1.09 ± 0.65	3.15 ± 1.18	4.89 ± 1.27	1.78 ± 0.38
*7*	*C9-1*	*2-Nonanone^2^*	1.88 ± 0.24	2.12 ± 0.34	3.05 ± 0.78	1.23 ± 0.08
8	C9-2	2-Nonanol^2^	0.32 ± 0.12	0.39 ± 0.11	0.32 ± 0.08	0.24 ± 0.07
9	FU-2	**2-Heptyl furan^2^**	0.11 ± 0.03 a	0.30 ± 0.05 b	0.43 ± 0.12 b	0.11 ± 0.01 a
10	MT-1	p-Menth-2-en-7-ol^2^	0.23 ± 0.01	0.20 ± 0.03	0.23 ± 0.05	0.27 ± 0.03
11	*C11*	*2-Undecanone^2^*	0.16 ± 0.03	0.32 ± 0.06	0.59 ± 0.18	0.10 ± 0.03
12	*ST-1*	***α–Bergamotene^2^***	0.16 ± 0.05 a	1.25 ± 0.36 b	2.48 ± 0.88 b	0.06 ± 0.01 a
13	*ST-2*	*(E)-β-Farnesene^2^*	0.25 ± 0.06	0.49 ± 0.10	0.70 ± 0.19	0.17 ± 0.04
14	*6PP-1*	***6-Pentyl-2H-pyran-2-one^2^***	37.80 ± 23.90 a	59.30 ± 22.20 a	97.80 ± 38.70 a	1.26 ± 0.44 b
15	ST-3	β-Curcumene^2^	0.53 ± 0.07	0.68 ± 0.09	0.49 ± 0.16	0.33 ± 0.04
16	ST-4	α-Farnesene^2^	0.09 ± 0.03	0.26 ± 0.03	0.28 ± 0.08	0.13 ± 0.01
17	ST-5	β-Sesquiphellandrene^2^	0.26 ± 0.07	0.25 ± 0.05	0.33 ± 0.09	0.19 ± 0.01
18	*6PP-2*	***6-Pent-1-enyl-2H-pyran-2-one^3^***	0.97 ± 0.55 a	1.78 ± 0.48 a	2.37 ± 0.90 a	0.04 ± 0.02 b
19	OT-2	Unknown [77.132. 175]	0.45 ± 0.12	0.34 ± 0.02	0.32 ± 0.09	0.27 ± 0.09
20	ST-6	Zingiberenol^2^	0.30 ± 0.05	0.41 ± 0.15	0.26 ± 0.08	0.14 ± 0.03
21	*DT-1*	***Unknown diterpene^3^***	2.57 ± 1.30 ab	4.04 ± 1.19 b	11.50 ± 3.85 b	0.62 ± 0.09 a
22	DT-2	Unknown diterpene^3^	0.00 ± 0.00	0.04 ± 0.01	0.27 ± 0.13	0.00 ± 0.00
23	DT-3	**Unknown diterpene^3^**	0.61 ± 0.33 a	0.92 ± 0.29 a	0.00 ± 0.00 b	0.11 ± 0.01 c
24	DT-4	**Unknown diterpene^3^**	0.00 ± 0.00 a	0.00 ± 0.00 a	2.75 ± 0.95 b	0.00 ± 0.00 a
25	DT-5	Unknown diterpene^3^	0.00 ± 0.00	0.00 ± 0.00	0.10 ± 0.05	0.00 ± 0.00
26	*DT-6*	***Unknown diterpene^3^***	0.21 ± 0.07 ac	0.46 ± 0.08 ab	0.86 ± 0.28 b	0.09 ± 0.00 c
27	DT-7	Unknown diterpene^3^	0.32 ± 0.05	0.23 ± 0.04	0.50 ± 0.24	0.18 ± 0.01
28	DT-8	Unknown diterpene^3^	0.25 ± 0.04	0.16 ± 0.03	0.25 ± 0.09	0.13 ± 0.01
29	DT-9	Unknown diterpene^3^	5.91 ± 0.60	5.40 ± 0.80	5.96 ± 1.12	3.67 ± 0.26
		Total	106.02 ± 36.23	123.79 ± 34.21	183.61 ± 61.98	55.73 ± 2.59

*^*1*^OT, Other; C7, C8, C9, and C11 indicate the number of carbon atoms in the compound; FU, Furan; MT, Monoterpene; ST, Sesquiterpene; DT, Diterpene; 6PP, Pyranone derivative. ^*2*^Compound identification based on mass spectrum and retention index. ^*3*^Compound identification based on mass spectrum only. Compounds italicized are those whose levels detected per strain matched to the fungistasis phenotype shown in Figure 5. Compounds in bold differ in their abundances with different letters indicating statistically significant differences (p < 0.05).*

**FIGURE 6:**
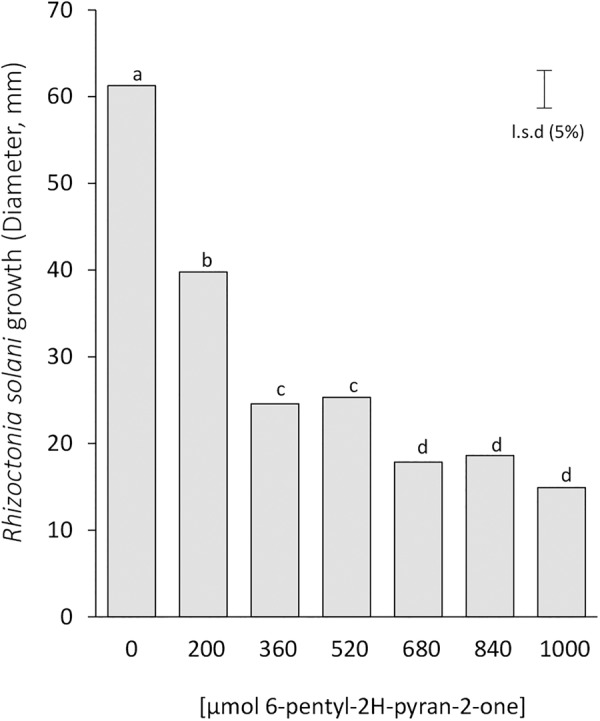
Inhibition of *Rhizoctonia solani* growth by 6-pentyl-2H-pyran-2-one in the inverted plate assays. The pathogen was incubated for 2 days at 25°C above paper disks to which 6-pentyl-2H-pyran-2-one (6PP-1) was applied at the time of pathogen inoculation. The least significant difference (LSD 5%) is indicated by a bar; different letters on top of bars indicate statistically significant differences of R. solani growth among 6PP-1 concentrations. The experiment was done twice with similar results.

Based on this indication, we tested the impact of the 6-pentyl-2H-pyran-2-one on the plant pathogens *R. solani* by a dose-dependent growth curve for the commercially available 6-pentyl-2H-pyran-2-one (6PP-1). This compound significantly inhibited the vegetative growth of *R. solani* in a dose-response manner with the highest inhibition rate up to 78% with respect to the growth of the untreated control (Figure 6). Interestingly, the results also indicated that the fungistatic effect appeared to reach a plateau (680 μmoles), above which the further increases in 6PP-1 amounts did not further reduce mycelial growth. These data support both the less pronounced plant growth and the growth inhibition of *R. solani* and *S. sclerotiorum* observed by the mutants Δ*nox1* and Δ*noxR* (high producers of 6PP-1), and the lack of effects for Δ*nox2* (low producer of 6PP-1) (Figures 4–6). However, these results do not exclude a possible simultaneous effect of the other indicated VOCs on the less pronounced (inhibitory) plant growth and on the fungistatic activity in *T. atroviride* (Figures 4, 5 and Table 1). For instance, the other molecules synthesized at overall lower levels by *T. atroviride*, but with significant differences among the strains (e.g., α–bergamotene and unknown diterpenes -1 and -6; Table 1), also demonstrated a detection pattern compatible to the plant growth and fungistatic results shown in Figures 4, 5.

## Discussion

In this study, we assessed potential roles of two ROS-generating, NADPH oxidase genes, *Nox1* and *Nox2* and the regulatory protein *NoxR*, using single-gene mutations in a strain of *T. atroviride* as the model system. Our dataset suggests that NoxR and Nox1 proteins are relevant for cell metabolisms that deal with growth, development, reproductive differentiation, and common biotic interactions with plants and other fungi. These two tightly interconnected genes appear to be the ones involved in the regulation and signaling of those cellular processes, confirming a series of previous studies (Hernandez-Oñate et al., 2012; Hernandez-Oñate and Herrera-Estrella, 2015). On the other hand, to our knowledge, this is the first report showing a functional activity for Nox2 in *Trichoderma*, related to an inducible metabolic response linked to production/emission of volatiles with effects on plant-growth and on fungistatic activity (at a distance).

The fact that the phenotypes of Δ*noxR* and Δ*nox1* were similar throughout this study supports the notion that *NoxR* and *Nox1* participate in the same cellular functions, as previously observed during differentiation by mechanical injury (Hernandez-Oñate et al., 2012; Medina-Castellanos et al., 2014). Most of the results are consistent with the fact that NoxR is a necessary component for Nox1 and Nox2 regulation in fungi (Scott and Eaton, 2008; Tanaka et al., 2008; Scott, 2015; Marschall and Tudzynski, 2016) and that *noxR* has been found in all fungal genomes containing orthologs to *Nox1/A* (Takemoto et al., 2007). As additional support for this view, identical phenotypes for *NoxR* and *NoxA* fungal mutants were also observed to be associated with changes from an endophytic to a pathogenic interaction of *E. festucae* with perennial ryegrass (Takemoto et al., 2006) and generation of ROS through Nox1 and NoxR is essential for injury-induced conidiation (Hernandez-Oñate et al., 2012). In other fungal species, *Nox1/A* orthologs are critical for mycelial growth, conidiation, differentiation of sexual fruiting bodies, defense and antagonism (Lara-Ortiz et al., 2003; Tanaka et al., 2006; Cano-Dominguez et al., 2008; Montero-Barrientos et al., 2011; Yang and Chung, 2012; Marschall and Tudzynski, 2016). These NoxA-related functions are consistent with our results (Figures 1, 2, 4), thereby reinforcing the current view of a major role for this NADPH oxidase in the fungal kingdom. In turn, Nox2 is found in most fungi containing *Nox1*, showing partially overlapping cellular tasks in some species (Egan et al., 2007; Yang and Chung, 2013; Wang et al., 2014).

In *T. atroviride*, Nox1 and Nox2 seem to perform different roles, which is a feature also found in other fungi (Tanaka et al., 2006; Egan et al., 2007; Cano-Dominguez et al., 2008; Ryder et al., 2013; Marschall and Tudzynski, 2016). The *T. atroviride* Δ*nox2* behaved mostly identical to WT in morphology when exposed to all stressors tested. In contrast with the loss of function for Δ*nox*R and Δ*nox*1, the absence of *nox2* did not interfere with the common qualitative responses of the fungus to these stresses. However, when assessing quantitative differences in mycelial growth among strains (Figure 1B), Δ*nox2* differed from WT for the oxidative and membrane stressors, similarly to Δ*noxR* and *Δnox1*; this suggests the participation of the three NADPH oxidase genes in the response to these stresses. Congo Red and Calcofluor White are two cell wall polysaccharide binding fluorochromes that affect nascent glucan (CR) and chitin (CW) chains, respectively and therefore cell wall integrity (Roncero and Duran, 1985; Nodet et al., 1990). Interestingly, for the cell wall stressor CW, Δ*nox2* was the only mutant with significantly less mycelial growth, which suggests a possible specific role of Nox2 in this particular stress response.

The quantitative conidiation patterns observed for the WT and mutants suggest strong metabolic effects of the various stressing agents on this trait. Conidiation was overall fully suppressed by osmotic stresses (NaCl and sorbitol), suggesting that hyperosmotic stress activates or inhibits mechanisms which directly or indirectly affect fungal conidiation program. Oxidative (menadione) and cell-wall (CR and CW) stresses severely decreased conidiation, which was otherwise only mildly affected by membrane stresses (SDS) (Figure 1B). For these treatments, the results suggest that Nox2 apparently modulates conidiation on a somehow repressive mode during cell wall or membrane stresses, since this trait was significantly higher for this mutant, i.e., when the gene function was knocked-out. On the other hand, Nox1 seems to be involved in alleviating the cell-membrane stress, thereby helping conidiation, as a stronger decreasing effect on spore production was observed for its mutant (Figure 1B). Taken together, the results suggest that NADPH oxidase activity is somehow related to reproductive metabolism in *T. atroviride*, being required to modulate its response to different types of stresses. As discussed earlier, the differentiation of reproductive structures in fungi is subjected to regulatory effects of ROS (Lara-Ortiz et al., 2003; Aguirre et al., 2005; Cano-Dominguez et al., 2008; Wang et al., 2014; Scott, 2015). Similar results were found in the saprophytic fungus *Podospora anserina*, as *PaNox*1 disruption reduced sexual fruiting body formation, whereas *PaNox*2 deletion blocked ascospore germination (Malagnac et al., 2004). Further experiments are required to test whether conidia produced by Δ*nox2* are as viable as those produced by the WT under the same stress conditions.

Biolog Plates^TM^ experiments for studying sole-carbon-source utilization profiles provided an alternative and interesting manner to obtain a ‘metabolic fingerprinting’ of the strains under study, when all individual nutrients were assessed simultaneously. The results revealed specific patterns of growth and conidiation for the WT and mutant strains, based on their consumption of the same collection of C sources (Supplementary Figure S2). The more closely related profiles of Δ*noxR* and Δ*nox1* for both traits mostly corroborated our morphological results in Figure 1 and added support to the statement that the function of the corresponding proteins is more closely linked to each other than to Nox2. Moreover, the roles of Nox1 and NoxR in regulating metabolisms related to the conidiation trait appeared to be more critical than for mycelial growth in *T. atroviride*, as reported in other systems (Cano-Dominguez et al., 2008; Wang et al., 2014; Scott, 2015). This can be concluded from the observation that the Δ*noxR* and Δ*nox1* conidiation levels were the lowest in the vast majority of C sources tested (Supplementary Figure S2B).

The Biolog Plates analysis revealed specific, subtle differences between WT and mutant strains, which suggests specific involvement of ROS in these metabolic systems. For example, all *Nox* mutants were compromised in their assimilation of sucrose (12 C) and fructose (6 C), but not of glucose (6 C) (Figure 2). Since these two monosaccharides are the products of sucrose cleavage, this suggests that the specific metabolism of fructose is affected by the loss of function of the three NAPDPH oxidases, which, in turn, is likely responsible for the reduction in growth observed for the three mutant strains in sucrose. Further experimental work is certainly warranted to underpin the regulatory framework of fructose metabolism via Nox proteins. The loss-of-function for each of the three *Nox* genes also had a reduction effect in the growth of the mutant strains on xylose (Figure 2). This suggests the involvement of these oxidases in either xylose catabolism or in its transport. Furthermore, D-xylose is a repressor or inducer of xylanase expression in *Trichoderma reesei*, a regulation that is dependent on the concentration of the sugar, and also involves the carbon catabolite repression mechanism (Mach-Aigner et al., 2010). The D-xylose to xylitol conversion involves a D-xylose reductase (Xyl1) which requires NADPH (Hong et al., 2014). This could suggest that the reservoirs of this metabolite can be reduced in the NADPH oxidase mutants and consequently affecting utilization of this sugar. The three *Nox* mutants are also partially compromised in their utilization of galactose (Figure 2). The oxidoreductive pathway of galactose to fructose involves at least three redox reactions, in which two are dependent on NADPH. The second step in the D-xylose catabolism from *T. reesei* involves NAD-xylitol dehydrogenase (Mach-Aigner et al., 2010).

The statistically significant differences found between WT, Δ*nox1* and Δ*noxR* in both antagonism systems (Figure 3) suggest that these proteins are involved in the fungus-fungus interaction. This result is compatible with the tight connection suggested between NoxR and Nox1. Moreover, the dual-culture confrontation results for both pathogens suggest that the product of the *Nox2* gene may have a minor involvement in the final phenotype related to the interaction with other fungi. Based on phenotypes of the Δ*noxR* and Δ*nox1* mutants, two possibilities may be advanced: either (1) the observed effects were the result of a role for NoxR and Nox1 in regulation of the hyphal contact and interaction of *Trichoderma* with other fungi, as already indicated in other systems (Montero-Barrientos et al., 2011; Wei et al., 2016), or (2) the decrease in antagonism in these mutants was simply a pleiotropic effect of a somewhat lower speed of growth of Δ*noxR* and Δ*nox1* mutants in relation to WT (data not shown). Overexpression of *Nox1* in *T. harzianum* increased the expression of lytic enzyme encoding genes and other components involved in cell wall remodeling (Montero-Barrientos et al., 2011) which strengthens the first possibility.

The VOCs related to increases in plant biomass may most likely function as signaling molecules capable of activating hormones acting on plant growth and development pathways (Hung et al., 2013). A recent study has shown that *Trichoderma* species are capable of synthesizing and emitting a blend of VOCs with ability to promote increases in biomass, plant height and chlorophyll content, thereby improving photosynthetic rates (Lee et al., 2016; Nieto-Jacobo et al., 2017). Hence, the emission of VOCs with plant-growth promotion activity by *Trichoderma* seems to be a type of metabolism likely related to biotic interactions of microorganisms with plants in nature (Bitas et al., 2013; Schmidt et al., 2015). Recently, it has been shown that *A. thaliana* seedlings induce the biosynthesis of the volatile 6-pentyl-2H-pyran-2-one (6PP-1) in *T. atroviride* IMI206040 (the same isolate of the present study), thereby suggesting the existence of plant signals that act at a distance by triggering a VOC-emission response in the fungus (Garnica-Vergara et al., 2016). The interaction between *Trichoderma* spp. and plants has been widely reported (De Souza et al., 2008; Chaverri et al., 2011; Hohmann et al., 2011; Ding et al., 2012; Rosmana et al., 2016; Nogueira-Lopez et al., 2018). The correlation of the absence of *nox1* and *noxR* genes function in *T. atroviride* and the observed response of a partial loss of growth-promoting effect might be explained by emission of a different set of specific VOCs with plant-growth promoting ability. The fact that 6PP-1 was one of the two volatiles (the other being C7-1) found in higher proportional amounts in the blend of VOCs (Table 1) is consistent with previous findings, which have proposed this volatile as capable of interfering in plant growth (Garnica-Vergara et al., 2016). Strains that produce 6PP, such as *T. asperellum* IsmT5 (Kottb et al., 2015) and *T. atroviride* CBS 351.93 (Lee et al., 2016), show a negative impact on plant growth. Δ*noxR* and Δ*nox1* synthesized more 6PP-1 and 6PP-2 than the other strains, with their growth-promoting effects being significantly lower (Figure 4B). Taking together with their likely effects on inhibition of other fungi growth (Figure 5; see discussion next), the results suggest that these compounds at higher doses might be either responsible for the reduction in plant-growth stimulation or a direct inhibitory effect on growth of plant cells.

It is also important to consider that microorganisms can release their VOCs mixed with other inorganic compounds, such as CO_2_ which can have a direct impact on plant growth (Effmert et al., 2012; Piechulla and Schnitzler, 2016). In this study, we could disassociate the effect of fungal growth with the CO_2_ produced by *Trichoderma*, as additional experiments using Ba(OH)_2_ and charcoal allowed us to consistently observe the effect of *Trichoderma* VOCs and CO_2_ on the biomass increase of *A. thaliana* (Supplementary Figure S3). The Nox-mutants supplemented with the respective functional genes confirmed that the strains recovered the WT phenotype, thereby confirming the effects associated with gene loss-of-function (Figure 4). These results, together with those from the volatile-mediated fungistasis experiments, interestingly suggest that *NoxR* and *Nox1* could be players (or modulators) in a repressive regulatory mode related to emissions of VOCs with inhibitory effects on cell growth from other organisms; when their function is eliminated, the inhibitory/fungistatic VOC synthesis/emission is increased. This view is compatible with the possibility that *T. atroviride* may be constantly “sensing” the presence of other competitors (as in the paired-plate experiments), likely through signals that are also related to VOCs emission by other fungi and/or plants. The presence of a functional *Nox2* gene coupled with absence of either *Nox1* or *NoxR* was shown to be strictly connected to production of inhibitory/fungistatic VOCs in *T. atroviride* (Figures 4, 5). A reasonable explanation to account for these phenomena is that, unlike Nox1 and NoxR, Nox2 may have a role in regulating the production of VOCs, particularly for the 6-pentyl-2H-pyran-2-one (6PP-1), 6-pent-1-enyl-2H-pyran-2-one (6PP-2), β-bergamotene (ST-1) and unknown diterpenes -1 and -6 (Table 1). Since the 6PP-1 compound is widely recognized as a fungistatic volatile produced by *Trichoderma* strains (Tarus et al., 2003; Reithner et al., 2005), the use of a commercial form of 6PP-1 helped to confirm that this metabolite is indeed at least one of the volatiles in the blend of *T. atroviride* IMI206040 VOCs that is responsible for the fungistatic activity over *R. solani* (Figure 6). Taken together, our results indicate that the VOCs causing fungal/plant inhibitory effects and enhanced plant growth, though co-produced, are very likely not the same. It is noteworthy that, although Nox2 appears to regulate VOCs, the synthesis of these metabolites by *T. atroviride* could also be triggered by VOCs emitted by plants, through a Nox2-independent mechanism. Further experiments are required to sort out this possibility.

Considering all the results of this study together, we suggest the following regulatory scenario for the NADPH oxidases of *T. atroviride*: NoxR, as a putative regulatory protein based on sequence similarity to others previously characterized (Takemoto et al., 2006), is closely associated with Nox1 (Takemoto et al., 2006; Hernandez-Oñate et al., 2012). As observed in other fungal models, both genes likely have direct participation in development and differentiation (Aguirre et al., 2005; Egan et al., 2007; Cano-Dominguez et al., 2008; Ryder et al., 2013), based on important primary metabolic processes (Figures 1, 2), as well as on other interactive mechanisms with the biotic environment (Figures 3–5). On the other hand, Nox2 seems to regulate different pathways from Nox1 and NoxR, being involved mostly in secondary metabolism that deals, at least, with (i) positive regulation of CW-dependent cell-wall stress (Figure 1B), (ii) negative regulation of conidiation under membrane and cell wall stresses (Figure 1C), and (iii) synthesis of VOC(s) with plant-cell growth inhibition and fungistatic abilities (Figures 4, 5 and Table 1). The ability of emitting substances (such as VOCs with inhibitory action on cell growth) that act at a distance by inhibiting other competitive organisms would expectedly confer advantage to the emitter toward a preferential use of the resources for growth and development. We believe this theoretical framework can serve as a reasonable basis for further hypothesis-driven research aiming at revealing specific cellular mechanisms and pathways regulated by Nox2 production of ROS.

## Author Contributions

VC-M, MN-J, DK, JK, FP-A, EZJ, MR, and AM-M performed the experimental work. AM-M, LL, MR, JH, and EZJ designed the experiments. VC-M, AM-M, LL, JS, MR, EZJ, and AH-E discussed and interpreted the results. VC-M, LL, MR, EZJ, and AM-M designed the research. AM-M, LL, AS, JH, and AH-E contributed to chemicals and scientific advice. VC-M, LL, JS, and AM-M wrote the manuscript. All authors reviewed the final version of the manuscript.

## Conflict of Interest Statement

The authors declare that the research was conducted in the absence of any commercial or financial relationships that could be construed as a potential conflict of interest.

## Footnotes

1 https://genome.jgi.doe.gov/Triat2/Triat2.home.html
