# Supplementary material for: The NADPH Oxidases Nox1 and Nox2 Differentially Regulate Volatile Organic Compounds, Fungistatic Activity, Plant Growth Promotion and Nutrient Assimilation in Trichoderma atroviride
Source: Front Microbiol. 2019 Jan 23;9:3271. doi: 10.3389/fmicb.2018.03271 (PMC6351448; doi:10.3389/fmicb.2018.03271)

NADPH oxidases Nox1 and Nox2 differentially regulate volatile organic compounds, fungistatic activity, plant growth promotion and nutrients assimilation in *Trichoderma atroviride*

Valter Cruz Magalhães[1,2], Maria Fernanda Nieto-Jacobo[1], Eline van Zijll de Jong[1], Michael Rostás[1], Fabiola Padilla-Arizmendi[1], Diwakar Kandula[1], Janaki Kandula[1], John Hampton[1], Alfredo Herrera-Estrella[3], Johanna M Steyaert[1,4], Alison Stewart[5], Leandro Lopes Loguercio[2,*] Artemio Mendoza-Mendoza [1,*]

[1] Bio-Protection Research Centre, Lincoln University, Lincoln, New Zealand

[2] Dept. Biological Sciences (DCB), State University of Santa Cruz (UESC), Rod BR 415, Km 16, Ilhéus-BA, 45662-900, Brazil.

[4] Langebio, Cinvestav Irapuato, Mexico

[4] Lincoln Agritech Ltd, PO Box 69133, Lincoln, Christchurch 7460, New Zealand.5

[5] The Foundation for Arable Research (FAR), Innovation Park, 185 Kirk Road, Templeton, Christchurch 7678, New Zealand.

Running title: VOCs and nutrient assimilation are differentially regulated by NADPH oxidases in *Trichoderma*

*** Corresponding Authors:**

Artemio Mendoza Mendoza
artemio.mendoza@lincoln.ac.nz

Leandro Lopes Loguercio
leandro@uesc.br

Keywords

Trichoderma, plant growth promotion, volatile organic compounds, Nox1, Nox2, NoxR, reactive oxygen species

Supplementary Figure S1. A. PCR for confirming the integration of the complementation construct into the ΔNox1, ΔNox2 and ΔNoxR deletion mutant strains. PCR from independent transformants was conducted using internal primers which amplify the open reading frame of the genes integrated in the deletion mutants. B. assay of mechanical injury in Nox1 and NoxR and the corresponding complementing strains to demonstrate the recovery of one of the phenotypes of the lack of these genes in *T. atroviride*.

A

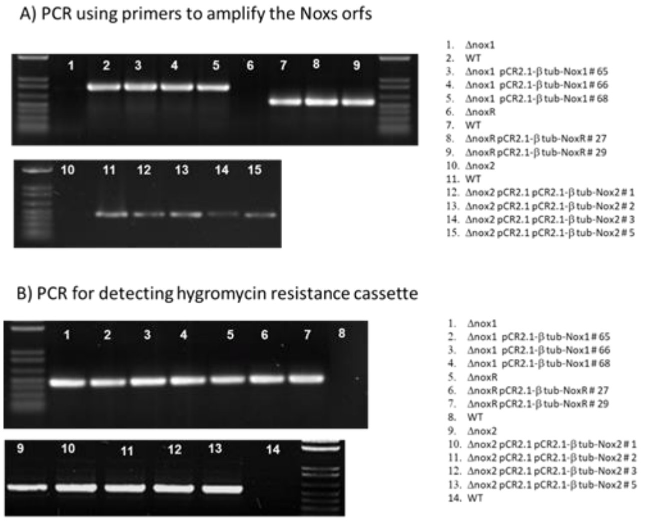

B

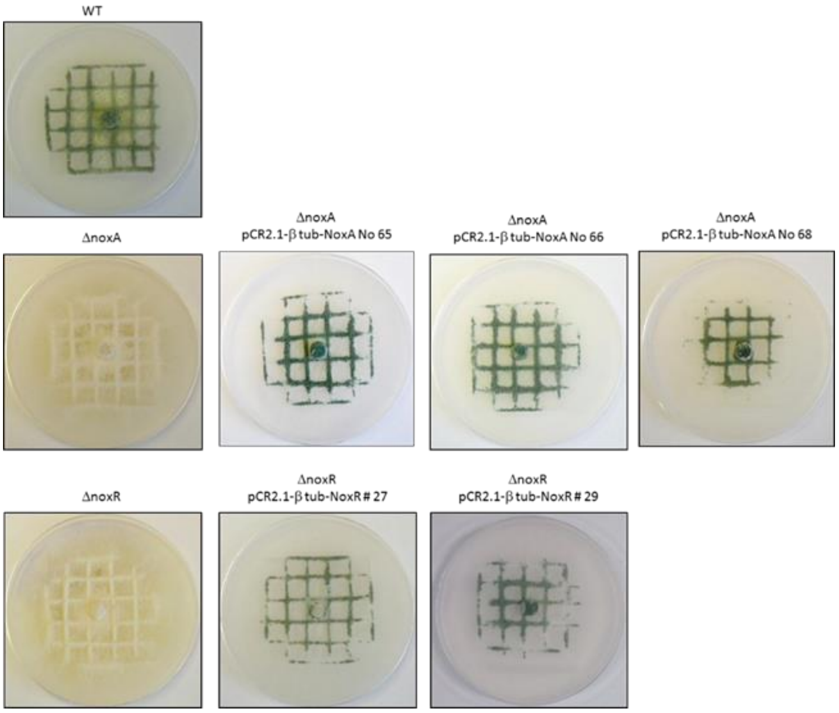

Supplementary Figure S2A. Comparative carbon sources utilization profiles of the Δ*nox1*, Δ*nox2*, Δ*noxR* mutants and *wt* of *T. atroviride*. The cluster analyses are shown as colored mosaic attached to dendrograms. (A) Results of growth were assessed after 72 h of incubation at 25 ºC, based on OD_{750}. Biolog Phenotype Microarray System™ was used, with the four strains grown at 95 carbon sources using FF Plates.

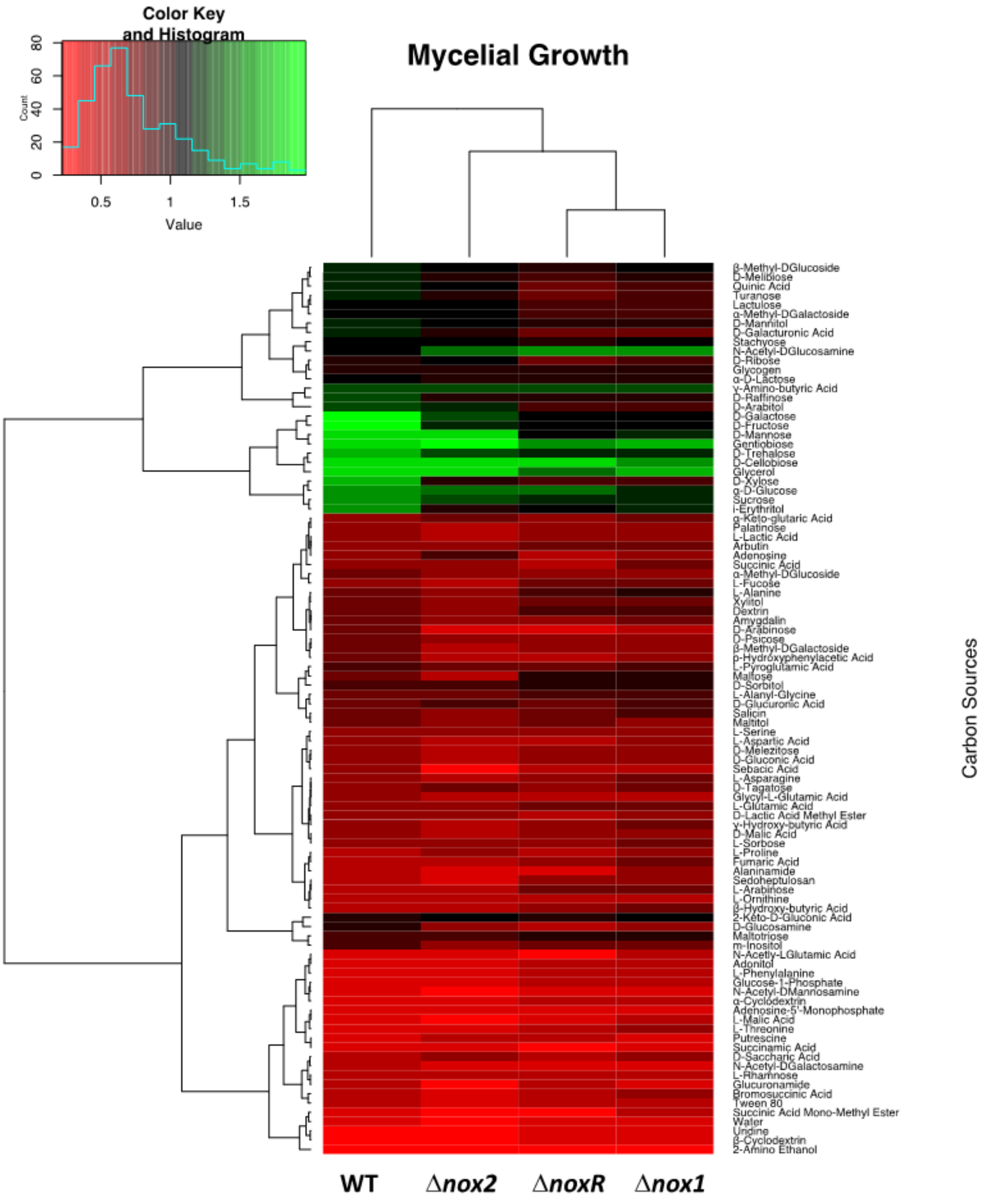

Supplementary Figure S2B. Comparative conidiation of WT, Δnox1, ΔnoxR and Δnox2 strains after 5 d of grown on 95 carbon sources using FF Plates from Biolog Phenotype Microarray System™. The color code indicated from red (no conidiation) to pale green (abundant conidiation)

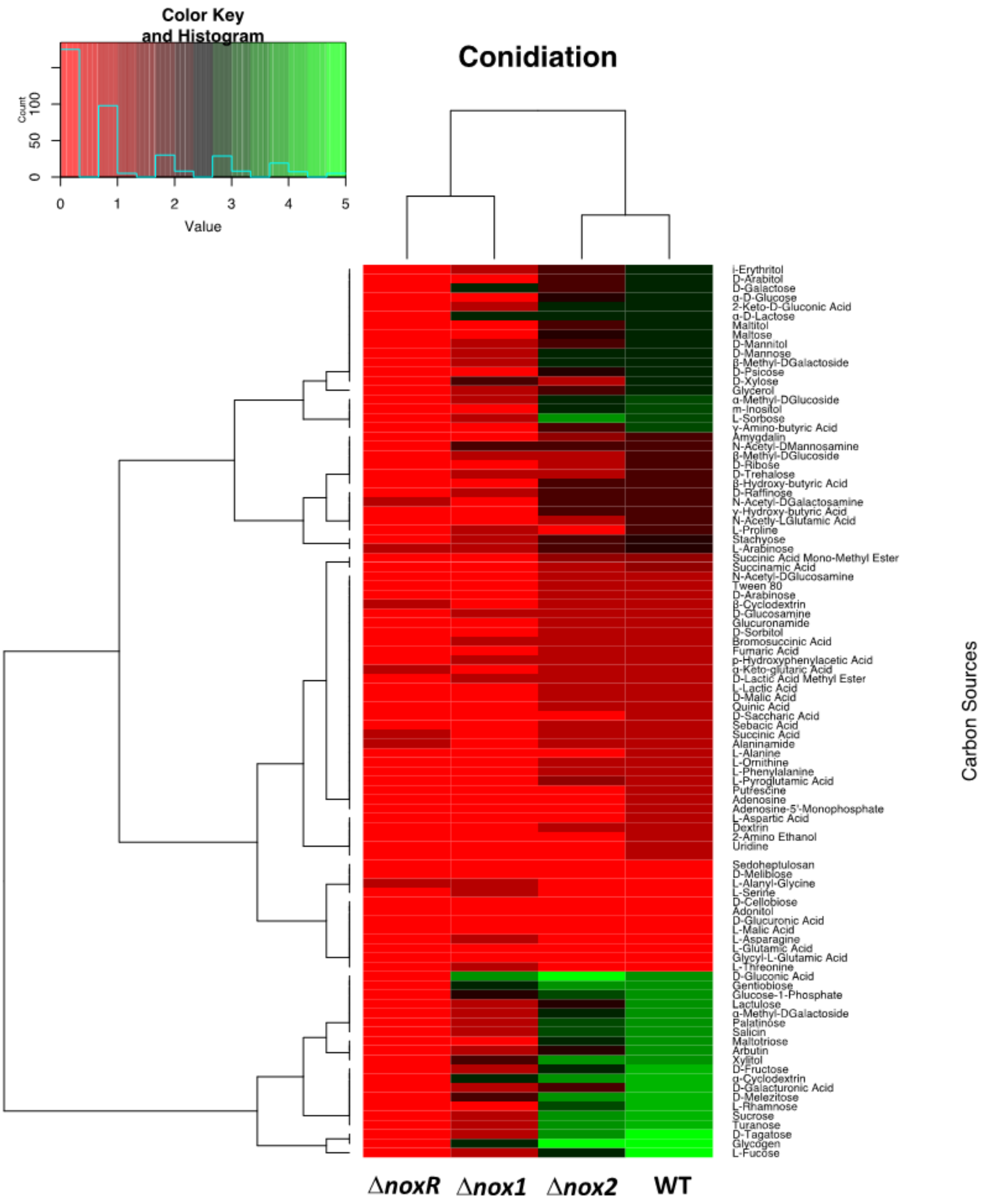

Supplementary Figure S3. Roles of CO_2 and *Trichoderma* VOCs in plant growth promotion. The effect of CO_2 was decreased/suppressed by addition of 100 mM of $Ba(OH)_2$ (bottom left), leaving the system under the VOCs action. The effect of *Trichoderma* VOCs was decreased/suppressed by addition of activated charcoal (bottom right), leaving the system under CO_2 action.

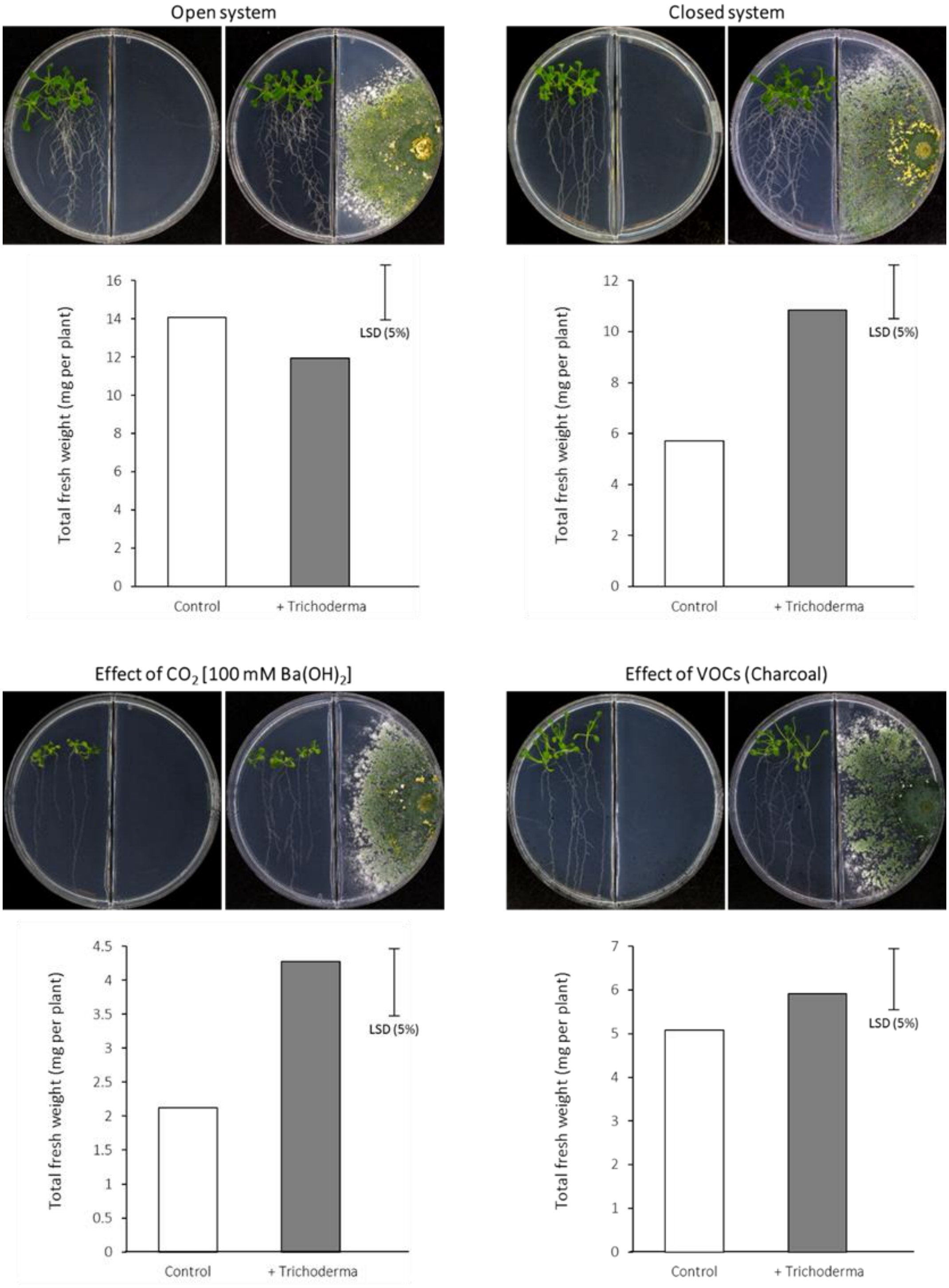

Supplement: Supplementary file 1 [file Presentation_1.pdf]
